# Simulations of time harmonic blood flow in the Mesenteric artery: comparing finite element and lattice Boltzmann methods

**DOI:** 10.1186/1475-925X-8-23

**Published:** 2009-10-02

**Authors:** Lilit Axner, Alfons G Hoekstra, Adam Jeays, Pat Lawford, Rod Hose, Peter MA Sloot

**Affiliations:** 1 Section Computational Science, Laboratory for Computing, System Architecture and Programming, Faculty of Science, University of Amsterdam, Science Park 107, 1098 XG Amsterdam, The Netherlands; 2 Department of Medical Physics and Clinical Engineering, University of Sheffield I Floor, Royal Hallamshire Hospital, Glossop Road, Sheffield, S10 2JF, UK

## Abstract

**Background:**

Systolic blood flow has been simulated in the abdominal aorta and the superior mesenteric artery. The simulations were carried out using two different computational hemodynamic methods: the finite element method to solve the Navier Stokes equations and the lattice Boltzmann method.

**Results:**

We have validated the lattice Boltzmann method for systolic flows by comparing the velocity and pressure profiles of simulated blood flow between methods. We have also analyzed flow-specific characteristics such as the formation of a vortex at curvatures and traces of flow.

**Conclusion:**

The lattice Boltzmann Method is as accurate as a Navier Stokes solver for computing complex blood flows. As such it is a good alternative for computational hemodynamics, certainly in situation where coupling to other models is required.

## Background

Atherosclerosis is the most common cardiovascular disease that affects the arteries [1-6]. It commonly first develops at the origins of tributaries, bifurcations and at curvatures of the arteries, in areas which are associated with low or oscillatory wall shear stress. The complexity of the human vascular system and the time-harmonic character of blood flow make analysis and prediction of its behavior very difficult. To obtain an information of blood flow on the locations of these regions in the human cardiovascular system, numerical studies are essential.

The lattice Boltzmann method (LBM) was proposed two decades ago, and is now recognized as a well-established method in computational fluid dynamics [7,8]. It has been used to simulate fluid flow in a wide range of complex geometries such as porous media [9-11] or geometries from medical applications [12-19]. Several studies comparing LB and FE methods for transient flows have shown good agreement between, where LBM is less expensive in terms of memory consumption and uses comparable computational times [20,21]. However, in all these studies, whether in 2D or 3D geometries, the fluid flow was time-independent. Comparison of LB and FE methods for time-harmonic flows is lacking.

We have compared LB and FE simulation results for a systolic flow in a realistic 3D geometry. As an application, we have used blood flow in the abdominal aorta(AA) and one of the major abdominal branches, the superior mesenteric artery (SMA). The SMA takes origin from the anterior surface of the AA, distal to the root of the celiac trunk. Diffuse atherosclerotic disease is rare in the SMA its occlusion leads to the death of patients.

For quantitative comparison between the two methods we used the velocity and pressure profiles of the simulated flows. We also studied the details of flow characteristics such as vortex formation and traces of flow.

## Methods

### Lattice Boltzmann method

The lattice Boltzmann method is based on the discrete velocity Boltzmann equation. We have used the lattice Bhatnagar-Gross-Krook model (LBGK) [7,22] adapted to systolic flows [12]. All parameters are in lattice units and we assume *δx *= *δt *= 1. The lattice-BGK equation is then;

(1)

with **e**_*i *_the finite set of discrete velocities, *t *the dimensionless relaxation parameter, *f*_*i*_(**x**, **t**) the density distribution function and  the equilibrium distribution defined by

(2)

*w*_*i *_is a weighting factor,  the speed of sound, *ρ *the hydrodynamic density determined by

(3)

and **u **the macroscopic velocity determined by

(4)

*b *is the number of directions.

The viscosity *ν *of the fluid is determined by

(5)

We apply the three dimensional 19-velocity (D3Q19)model [8] for time harmonic flows [13].

### Finite element method

As described in Jeays et al. [23], a transient CFD model was constructed by morphing a parametric mesh constructed from simple geometric primitives. The advantage of this process is that it is easy to control the element size distribution mapped onto the original geometry. It is robust in operation, and is ideally suited to the generation of dynamic CFD meshes of arterial systems that are free from major pathology. An unstructured triangular mesh was generated for the CFD analysis using the ANSYS pre-processor. Flow boundary conditions were determined based on phase contrast MRI velocity measurements. FLOTRAN (ANSYS Inc.) was used as a FEM solver for simulating the 3D Navier-Stokes equations as (see Jeays et al. [23]).

## Results and Discussion

### Experiments and validation

**Figure 1:**
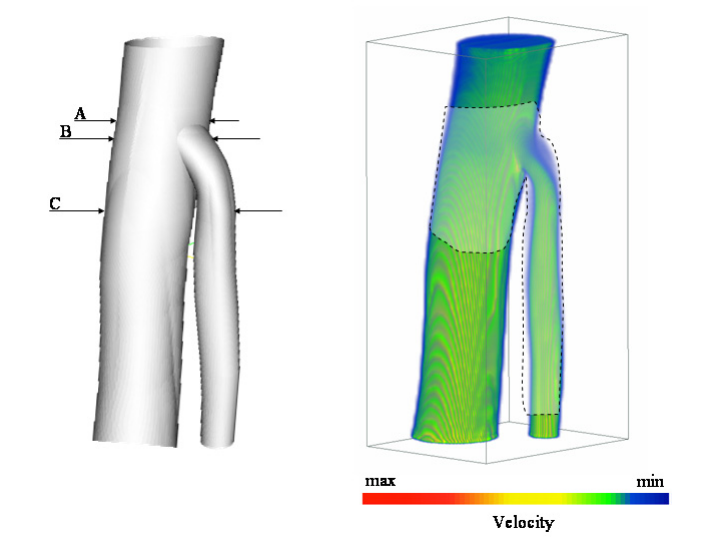
**The surface of the superior mesenteric artery (left) and a simulated velocity profile in it(right) at *Re *= 3300 with *α *= 11**.

The experiments were performed on the abdominal aorta together with the superior mesenteric arterial branch as shown in Fig. 1 (left).

The geometry was obtained from an Magnetic Resonance Imaging scan obtained from a healthy volunteer with full ethical approval. A triangular mesh was generated for use in the FEM solver [23]. The 3D mesh for FEM comprised 100465 nodes, with 650 nodes on the AA inlet, 295 on the AA outlet and 207 on the SMA outlet. From this mesh a voxel mesh was generated for LBM using a special 3D editing and mesh generation tool HemoSolve [24]. In this tool the parts of interest of geometry can be selected and can be enhanced with inlet and outlet layers on its end-points. Hemosolve has sufficient functionality to mimic a real surgical procedure. The generated voxel mesh from FEM triangular mesh was comprised of a total of 74468 fluid nodes with 694 nodes on the AA inlet, 316 on the AA outlet and 224 on the SMA outlet. For these simulations the walls of geometries are considered rigid as the effect of the elasticity is proven to be less than 3% (see ref. [23]).

The following flow parameters were applied in FEM: the density of the blood *ρ *was 1000 *kgm*^-3^, Newtonian viscosity *μ *was 4 * 10^-3 ^*Pa.s*, the cardiac cycle duration *T *was 0.86 *s *with 5 *ms *time-steps *δt *and the blood was considered incompressible. The maximum velocity *u *of 0.8 *m/s *at a peak systole in the aorta, combined with the diameter *D *of 0.0165 *m*, gave a maximum Reynolds number (Re) of 3300 [23]. In order to apply the same flow condition in the LBM solver we converted all parameters into dimensionless numbers. From the FEM parameters we computed a Womersley number of *α *= 11.14. *α *is the ratio between the time-harmonic flow frequency and viscous effects and is defined as .

Using these two constraints (*Re *= 3300 and *α *= 11.14) and aiming at no more than 10% simulation error (see [25]) we chose the maximum velocity *u*_*max *_= 0.08. Next, we minimized the execution time by applying the constraint optimization scheme, using the same procedure as described in [25] where we assume that the parametrization used for pipe flow was also applicable to the current geometry. This gave *T *= 17200, e.g. in physical units a *δt *= 5 * 10^-5 ^time step, while *D *= 30 lattice points. Finally from these parameters we derived *ν *= 0.00068.

We applied the same inlet/outlet boundary conditions as described in Jeays et al. [23]. Velocities (created using Womersley's solution [26]) were specified at the proximal AA opening, the pressure waveform (created using Westerhof's model [27]) was specified at the distal AA opening and a free-flow boundary condition was applied at the outflow of the SMA. We first transformed these given velocities into dimensionless values and then applied the velocity and pressure boundary condition as described in Ref. [28] at the proximal AA and distal AA openings respectively.

In this study, for LBM a bounce back on links (BBL) boundary condition is applied on the walls. We did not use more accurate boundary condition such as Bouzidi boundary conditions (BBC) [29]. These demand more elaborate computations especially for irregular geometries connected with the alpha parameter. From previous studies the order of both methods in terms of grid spacing are known [23,25]. Moreover, many formulations of solid boundary conditions are known and their influence on the accuracy on the flow fields is well understood [12,25]. We expect however that using BBC for LBM will only improve the results presented here, in the sense that either the accuracy will increase, or the choice of optimal simulation parameters changes drastically [25]. That is however not the topic of this paper.

### Comparison between two methods

After running simulations using both methods we compared the resulting velocity profiles at three different transverse planes (*A*, *B*, and *C*) as shown in Fig. 1.

**Figure 2:**
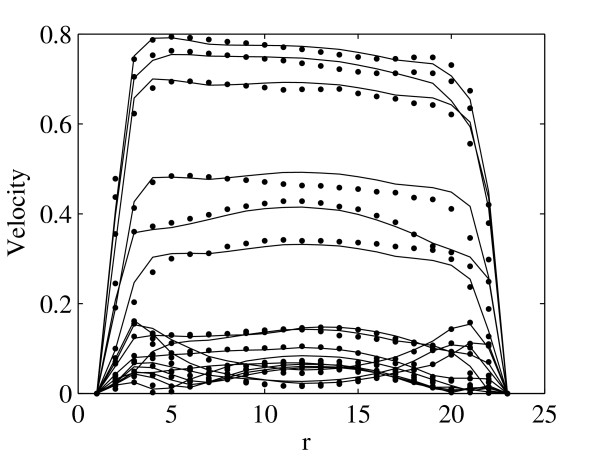
**Comparison of the velocity profiles between LBM (bullets) and FEM (solid lines) at the region *A *(see Fig. 1) at 0.0398 second intervals throughout one systolic period**. Velocities are presented in *m/s*.

**Figure 3:**
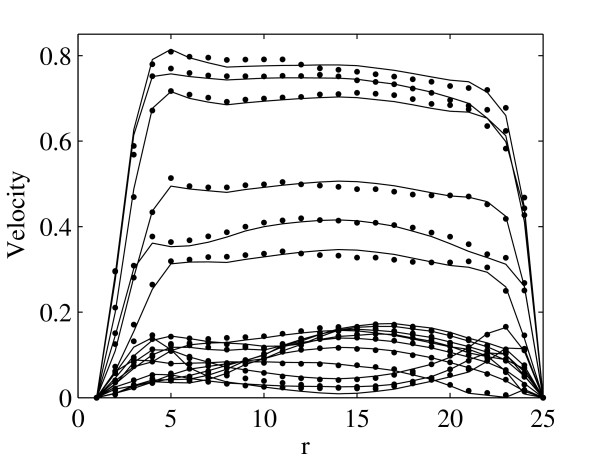
**Comparison of the velocity profiles between LBM (bullets) and FEM (solid lines) at the region *B *(see Fig. 1) at 0.0398 second intervals throughout one systolic period**. Velocities are presented in *m/s*.

**Figure 4:**
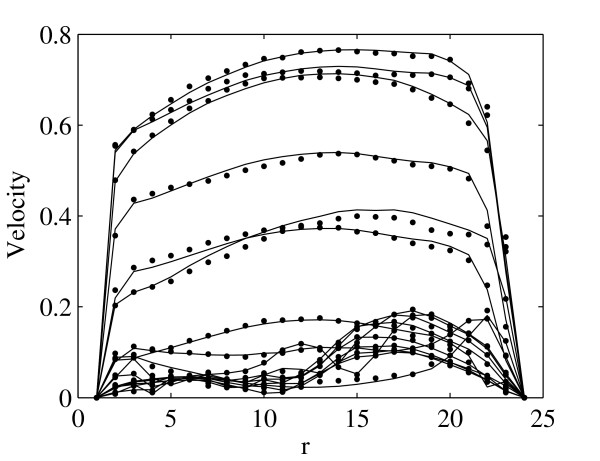
**Comparison of the velocity profiles between LBM (bullets) and FEM (solid lines) for the *AA *at region *C *(see Fig. 1) at 0.0398 second intervals throughout one systolic period**. Velocities are presented in *m/s*.

**Figure 5:**
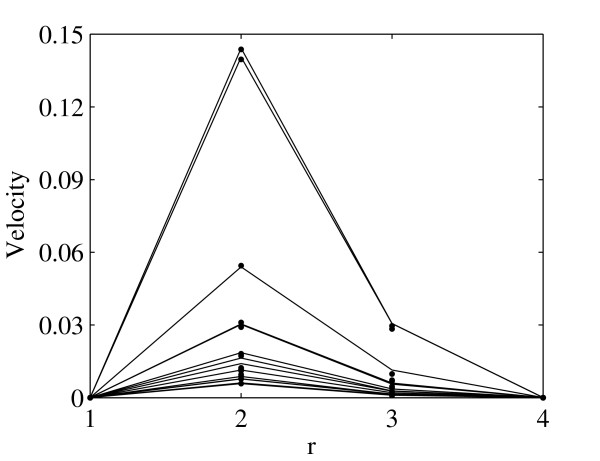
**Comparison of the velocity profiles between LBM (bullets) and FEM (solid lines) for the *SMA *at region *C *(see Fig. 1) at 0.0398 second intervals throughout one systolic period**. Velocities are presented in *m/s*.

The flow profiles at region *A *and *B *are shown in Fig. 2 and Fig. 3 correspondingly for region *C *in Fig. 4 and Fig. 5. The profiles on the cut planes are obtained at the lines aligned with the LBM grid, closest to the center. Here *r *is the location along the diameter *D*.

There is very good agreement between velocity profiles obtained by FEM and LBM. Moreover, the position of peak velocity in Fig. 2 and Fig. 3, e.g. before the bifurcation, is skewed to the left, the posterior aspect of the vessel, while in Fig. 4, e.g. after bifurcation, it is towards the right, the anterior aspect of the vessel. This indicates a spiraling of the flow in the AA.

**Figure 6:**
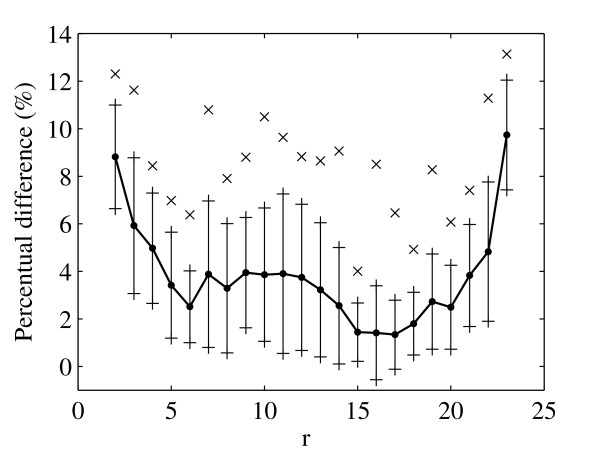
**Average percentage difference (line with bullets) between velocity profiles for both methods as a function of diameter (shown together with standard deviations (lines) and maximum differences (crosses)**.

**Figure 7:**
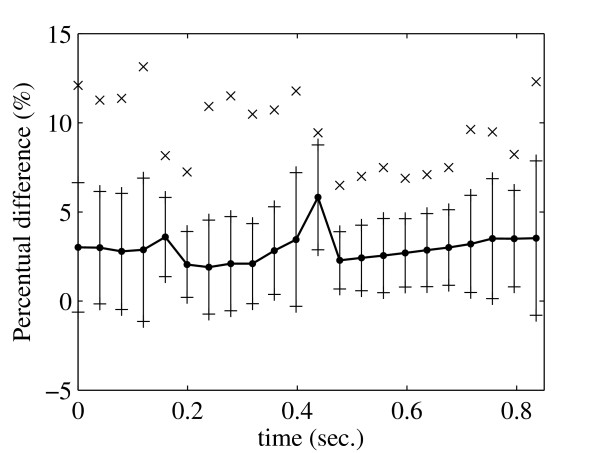
**Average percentage difference (line with bullets) between velocity profiles for both methods as a function of time (right) shown together with standard deviations (lines) and maximum differences (crosses)**.

In Fig. 6 and Fig. 7 we show the average percentage difference between the velocity profiles for both methods at the region *C *as a function of vessel diameter and time. These differences are computed from

(6)

where *a *is either diameter *D *or time *t*. The greatest average difference is observed close to the vessel walls (Fig. 6) and is approximately 10% while the maximum difference is not more than 13%. In Fig. 7 we see that the greatest average difference as a function of time is observed after peak systole and is about 6% while the maximum is about 13%.

**Figure 8:**
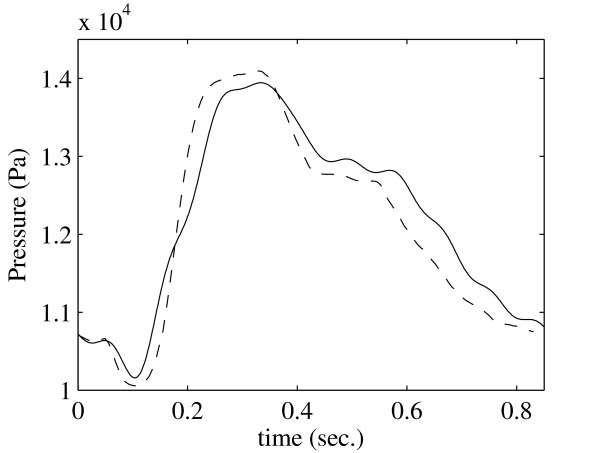
**Comparison of the average pressure profiles between LBM (dashed line) and FEM (solid line) at the outlet of the *SMA***. Pressure is presented in *Pa *over one systolic period.

**Figure 9:**
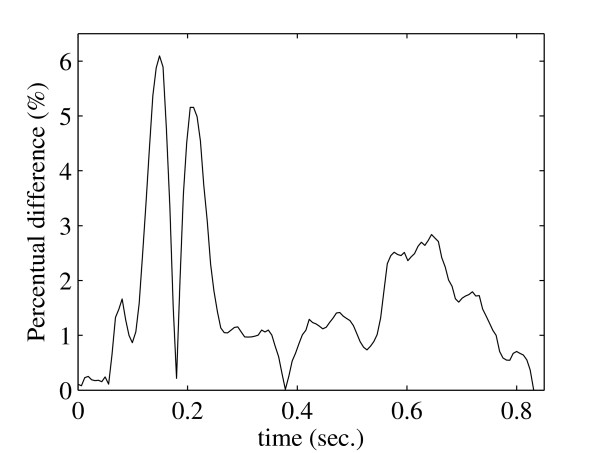
**Percentage difference between pressure profiles for LBM (dashed line) and FEM (solid line) at the outlet of the *SMA***.

Fig. 8 compares the pressure profiles at the outlet of the SMA. Here there is a very good agreement between both methods. As shown in Fig. 9 the average percentage difference is also quite small and maximum is 6.2%.

**Figure 10:**
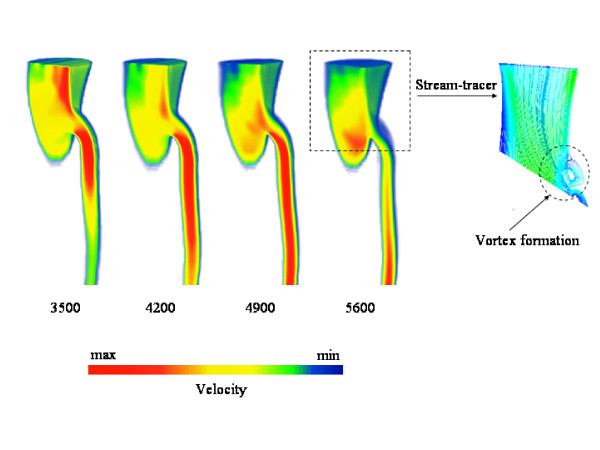
**Simulated velocity profiles depicted e.g. every 0.035 seconds during one systolic period and the stream-lines showing the vortex formation (right)**. Maximum and minimum velocities correspond to0.008*m/s *and 0.814*m/s *respectively.

Another interesting characteristic is that the flow in the SMA is directed from the bifurcation towards the outer wall, creating a vortex at the origin of the SMA in the anterior region (see Fig. 10). This vortex formation is due to the angle that the SMA forms with the AA [23].

In Fig. 10 we also show the velocity profiles at peak systole in the last cardiac cycle executed, when beat to beat convergence had been achieved, along the cutting plane shown in Fig. 1 (right). Here the expected flow behavior and the formation of a vortex at the top of SMA just below the outer wall can be clearly seen. For FEM the vortex first appeared at 0.26 seconds and disappeared at 0.56 seconds while, for LBM, it appeared at time-step 5200 and disappears at time-step 11200. Samples were taken every 100 time-steps and inspected visually. These results are completely identical if the *δt *= 5 * 10^-5 ^time-step is considered for the LBM simulation.

## Conclusion

We have validated the LBM for time-harmonic flows by comparing the simulation results with those obtained from numerically solving the Navier Stokes equations with FEM. As an experimental geometry we used the geometry of the human vascular system, the AA together with one of its major abdominal branches, the SMA. We compared velocity and pressure profiles of simulated time-harmonic blood flow for both methods and demonstrated a very good agreement. The maximum differences for velocity profiles were greatest next to the vessel walls. These were less than 10%. The maximum difference, as a function of time, was 6%. Moreover, the spiraling of the flow profiles in the AA and the time of vortex formation in SMA coincided with both methods.

The main purpose of this paper is to demonstrate that the LBM is a good alternative for computational hemodynamics. It is self evident that validation of a this new method against existing established methodologies (that is, using Navier-Stokes solvers) is an important step. We have drawn the Navier Stokes solution data for comparison from previous work by Jeays et al. [23]. We have chosen to focus our own efforts on demonstrating that the LB solver can reproduce the published Navier Stokes analysis by Jeays et al. [23] of a complex transient flow pattern in an arterial bifurcation, with the flow domain constructed from a medical image and the boundary conditions based on in vivo measurements. Some papers (e.g Ku et al [30], Ford et al. [31] have focused on the validation of Navier-Stokes solvers in the context of arterial flows, both by direct measurement in vivo and by measurement in anatomically-realistic experimental phantoms. In contrast, the emphasis in this manuscript is whether the numerical simulations produce an accurate solution of the problem posed. It is very well established that the most important determinant of any arterial flow, particularly in terms of the important local features such as flow separations, is the geometry. The whole point of the current work is to demonstrate that these local details are comparable between the LB solver and an established Navier Stokes solver. This paper confirms that the LBM represents a viable alternative to the Navier Stokes solvers for complex transient flow, in this case in an arterial bifurcation. It was quite remarkable that LBM stayed stable for such large *Re *= 3300 numbers. As for the computational expenses, LBM is less memory consuming and reported (see ref. [20]) to have computational times comparable to the FEM. With the modified simulator (see ref. [32]) we expect LBM to perform even faster.

With the increasing interest in using LBM for computational hemodynamics [15,17,18] this study shows that LBM can be considered to be an alternative as a solver for computational hemodynamics, producing results of equal quality to Navier Stokes solvers.

Another main difference between both methods lies in the mesh generation, which for LBM is obviously much easier. Moreover, suggested by Bernsdorf in his thesis [33], the LBM seems to be very well suited for the use in multiphysics models, for instance in blood clotting studies [34].

## Competing interests

The authors declare that they have no competing interests.

## Authors' contributions

LA and AJ carried out the simulations and drafted the manuscript. PL and RH suggested the case studied in this manuscript, and participated in data analysis. AGH conceived of the study and participated in its design and coordination, and finalized the manuscript. PMAS participated in the coordination. All authors read and approved the final manuscript.
